# Biocompatible Cryogel with Good Breathability, Exudate Management, Antibacterial and Immunomodulatory Properties for Infected Diabetic Wound Healing

**DOI:** 10.1002/advs.202304243

**Published:** 2023-09-04

**Authors:** Yang Li, Zifeng Yang, Qi Sun, Ruijun Xu, Renjie Li, Dingcai Wu, Rongkang Huang, Feng Wang, Yong Li

**Affiliations:** ^1^ School of Medicine South China University of Technology Guangzhou 510006 China; ^2^ Department of Gastrointestinal Surgery Department of General Surgery Guangdong Provincial People's Hospital (Guangdong Academy of Medical Sciences) Southern Medical University Guangzhou 510080 China; ^3^ Guangdong Cardiovascular Institute Guangdong Provincial People's Hospital Guangdong Academy of Medical Sciences Guangzhou 510080 China; ^4^ PCFM Lab, School of Chemistry Sun Yat‐sen University Guangzhou 510006 China; ^5^ Department of General Surgery (Colorectal Surgery) Guangdong Institute of Gastroenterology Biomedical Innovation Center Guangdong Provincial Key Laboratory of Colorectal and Pelvic Floor Diseases, The Sixth Affiliated Hospital Sun Yat‐sen University Guangzhou 510655 China

**Keywords:** antibacterial dressing, cryogel, immunomodulation, infected diabetic wounds, wound healing

## Abstract

Due to the complex microenvironment and healing process of diabetic wounds, developing wound dressing with good biocompatibility, mechanical stability, breathability, exudate management, antibacterial ability, and immunomodulatory property is highly desired but remains a huge challenge. Herein, a multifunctional cryogel is designed and prepared with bio‐friendly bacterial cellulose, gelatin, and dopamine under the condition of sodium periodate oxidation. Bacterial cellulose can enhance the mechanical stability of the cryogel by improving the skeleton supporting effect and crosslinking degree. The cryogel shows outstanding breathability and exudate management capability thanks to the interpenetrated porous structures. I_2_ and sodium iodides produced in situ by reduction of sodium periodate provide efficient antibacterial properties for the cryogel. The cryogel facilitates macrophage polarization from M1 to M2, thus regulating the immune microenvironment of infected diabetic wounds. With these advantages, the multifunctional cryogel effectively promotes collagen deposition and neovascularization, thus accelerating the healing of infected diabetic wounds.

## Graphical abstract

The simple synthetic, bio‐friendly and multifunctional bacterial cellulose/gelatin/polydopamine cryogel exhibits good biocompatibility, breathability, antibacterial, exudate management, and immunomodulatory properties, thus promoting the healing of infected diabetic wound.

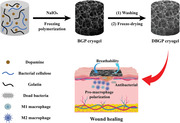

## 1 Introduction

The global prevalence of diabetes is gradually increasing. In 2021, it was estimated to be 10.5% among people aged 20–79 years, reaching 536.6 million, and diabetes‐related health expenditures reached 966 billion USD worldwide.^[^ ^1^ ^]^ It is expected to increase to 12.2% by 2045, with an estimated 783.2 million people affected, and the expenditures may reach to 1054 billion USD worldwide.^[^ ^1^ ^]^ Diabetic wounds are a common complication in diabetic patients, with a lifetime incidence of 19%−34%.^[^ ^2^ ^]^ Diabetic wounds are prone to persistent infections and poor healing.^[^ ^2^, ^3^ ^]^ If treatment is not timely or appropriate, amputation may even occur.^[^ ^3^ ^]^ Diabetic wounds seriously compromise patients' quality of life and health, and significantly increase the social medical costs.^[^ ^4^ ^]^ The effective diagnosis and treatment of diabetic wounds remain a challenging public health and clinical problem.

Persistent bacterial infection, impaired trophoblastic angiogenesis, disturbed local immune environment, excessive wound exudate, and improper wound management all give rise to persistent non‐healing of diabetic wounds.^[^ ^5^ ^]^ The early treatment of diabetic wounds involve disinfection, exudate control, immune environment improvement, angiogenesis promotion, infection control, blood glucose control, and surgical debridement.^[^ ^6^ ^]^ Studies have shown that regulation of macrophage polarization during the inflammatory phase plays a crucial role in diabetic wound healing.^[^ ^7^ ^]^ In early inflammation, classically activated M1 phenotype macrophages secrete large amounts of pro‐inflammatory cytokines to initiate and maintain inflammation, whereas M2 phenotype macrophages are associated with the regression or disappearance of chronic inflammation, promoting tissue repair and wound healing by secreting anti‐inflammatory cytokines.^[^ ^8^ ^]^

**Figure 1:**
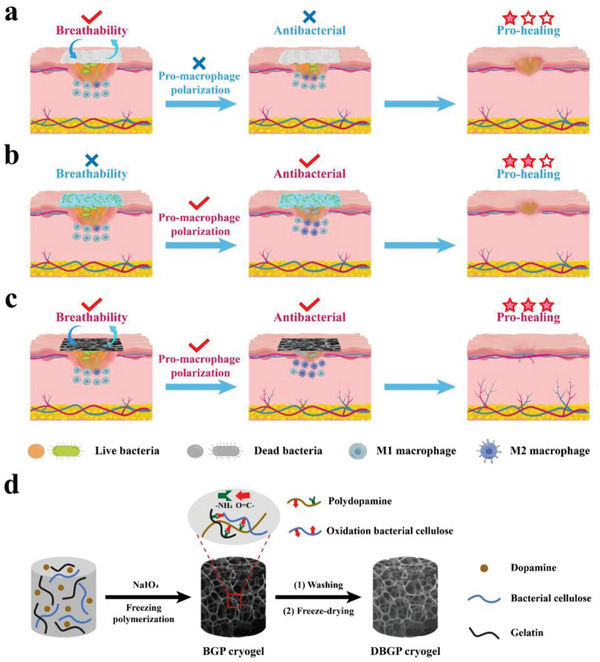
Schematics of the structure and performance of a) gauze dressing, b) multifunctional hydrogel dressing and c) DBGP cryogel dressing to promote healing of infected diabetic wounds: a) gauze dressing has good breathability, but does not have antibacterial and pro‐macrophage polarization properties to accelerate healing of diabetic wounds; b) multifunctional hydrogel dressing has antibacterial and pro‐macrophage polarization properties, but poor breathability, resulting in slow healing of infected diabetic wounds; c) DBGP cryogel dressing has excellent breathability, strong antibacterial and pro‐macrophage polarization properties, which effectively promotes healing of infected diabetic wounds; d) schematic of structure and synthesis of DBGP cryogel dressing.

Dressings are crucial for the local treatment of diabetic wounds. Traditional wound dressings, such as iodophor gauze, hydrocolloids, foam dressings, and silver‐containing dressings, cannot fully meet the requirements of diabetic wounds for absorbing exudate, keeping the wound moist, preventing infection, as well as allowing breathability. Moreover, the lack of immunomodulatory functions renders them insufficient for the entire healing process of diabetic wounds (**Figure** **1**a).^[^ ^6^, ^9^ ^]^ In recent years, multifunctional hydrogel dressings have been widely studied in infected diabetic wound healing. For example, antibacterial hydrogel dressings can inhibit bacterial multiplication and control infection.^[^ ^10^ ^]^ Hydrogels loaded with growth factors, such as vascular endothelial growth factor (VEGF), can promote angiogenesis and accelerate collagen deposition.^[^ ^11^ ^]^ Hydrogel dressings with endogenous immunomodulatory properties, such as hybrid hydrogel dressings based on glycyrrhizic acid (GA)^[^ ^12^ ^]^ or containing green tea derivatives,^[^ ^13^ ^]^ can induce macrophage polarization to promote rapid diabetic wound healing. However, hydrogel dressings may lead to wound maceration in diabetic wounds, because of excessive hydration, poor breathability, and small pore size. Meanwhile, the low survival and hindered migration of cells colonized inside the hydrogel often lead to irregular cell distribution,^[^ ^14^ ^]^ and the exchange of nutrients, oxygen, and metabolic waste in the hydrogel is also resistant (Figure 1b).^[^ ^15^ ^]^ In contrast, the cryogel with macroporous structure can effectively manage the exudate, diffuse nutrients, oxygen and biological molecules, keep metabolites and toxins away from cells, and promote cell crawling and proliferation.^[^ ^16^ ^]^ In addition, cryogel can also load functional components, achieving effective infection treatment.^[^ ^17^ ^]^ However, the current cryogel dressing still has many problems, such as the complex synthesis procedures^[^ ^17^, ^18^ ^]^ and the need to add toxic crosslinking agents (e.g., glutaraldehyde,^[^ ^19^ ^]^ EDC/NHS,^[^ ^20^ ^]^ ethylenediamine and glyoxal^[^ ^17^ ^]^). Furthermore, some cryogels use antibiotics and heavy metals to achieve antibacterial effects, which may lead to bacterial resistance and cumulative toxicity;^[^ ^18^, ^21^ ^]^ some cryogels have not been shown to induce macrophage polarization.^[^ ^18^, ^19^, ^20^ ^]^ Considering the complexity of diabetic wounds environment, the ideal cryogel dressing should be simple in synthesis, bio‐friendly, keeps the wound moist while absorbing excess exudate, and has antibacterial, breathable and immune regulating functions.

In this study, we have successfully prepared a novel class of cryogel by a facile one‐pot method using a mixture of bacterial cellulose (BC), gelatin (Gel), and dopamine (DA) as cryogel precursors in the presence of sodium periodate (SP) through oxidative polymerization reaction, Schiff base reaction and Michael addition reaction. Gel is used as a matrix material to mimic the extracellular matrix and provides a scaffold for cell adhesion and migration.^[^ ^22^ ^]^ The 3D fibrous structure and abundant hydroxyl groups of BC increase the framework support and crosslinking degree of the cryogel to enhance the mechanical properties,^[^ ^23^ ^]^ and avoid the use of chemical crosslinking agents such as EDC/NHS. DA functional group has the good adhesion performance^[^ ^24^ ^]^ as well as the function of promoting the polarization of M2 macrophages.^[^ ^25^ ^]^ I_2_ and sodium iodides produced in situ by reduction of sodium periodate provide efficient antibacterial properties for the cryogel and avoid the use of antibiotics, heavy metals, and other antibacterial agents. As a result, the dried bacterial cellulose/gelatin/polydopamine (DBGP) cryogel exhibits excellent biocompatibility, breathability, antibacterial, exudate management, and immunomodulatory properties. DBGP cryogel dressing could be used to treat infected wounds on the back of diabetic rats. The experimental results show that DBGP cryogel significantly promotes M2 macrophage polarization, collagen deposition, and neovascularization, thus accelerating the healing of infected diabetic wound (Figure 1c). We believe that this simple synthetic, bio‐friendly and multifunctional cryogel dressing could effectively promote the healing of infected diabetic wound, demonstrating potential clinical application prospects.

## 2 Results and Discussion

**Figure 2:**
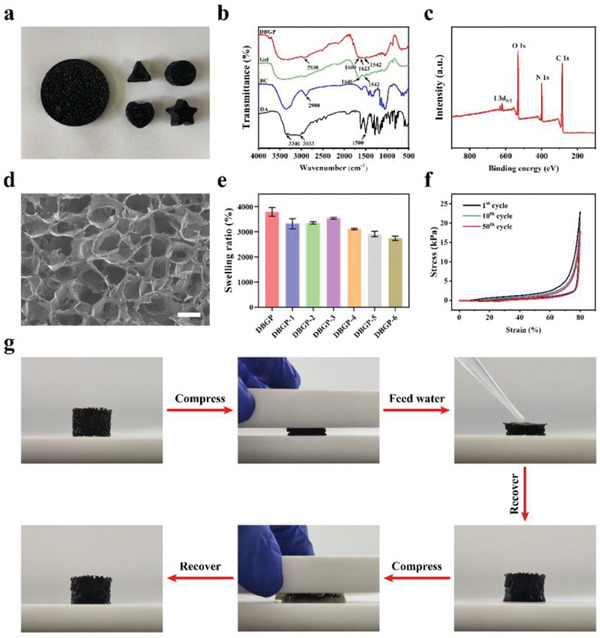
a) Digital photos of different shapes of DBGP cryogels; b) FT‐IR spectra of DA, BC, Gel and DBGP cryogel; c) XPS spectrum of DBGP cryogel; d) SEM image of DBGP cryogel (scale bar: 50 µm); e) swelling ratios of the cryogels; f) cyclic compression stress‐strain curves of DBGP cryogel; g) digital photos of a water‐triggered shape recovery property of DBGP cryogel.

Gel and DA are commonly utilized in gel preparation due to their biocompatibility and functionality.^[^ ^26^ ^]^ Gel with abundant amino and hydroxyl groups was used as the basic skeleton of gel.^[^ ^26^ ^]^ DA underwent oxidative polymerization in the presence of sodium periodate, reacted with the amino group on the Gel skeleton through the Schiff base and Michael addition reactions, and formed a hydrogen bond with Gel, eventually producing Gel/PDA (GP) cryogel.^[^ ^26^ ^]^ However, the crosslinking degree of GP cryogel is limited to some extent, so after thawing, GP cryogel is broken in the compression experiment and failed to reshape (Figure S1a, Supporting Information). Therefore, BC was introduced into GP cryogel to form BGP cryogel with higher compression stability. Firstly, we mixed BC, Gel, and DA aqueous solutions, added sodium periodate, and stirred to form a pre‐polymerization solution. Then we poured the solution into the mold to obtain BGP cryogel after the procedures of freeze polymerization at −20 °C and thawing at room temperature. Compared with GP cryogel, BGP cryogel is strong enough to withstand deep compression and recover completely after the external force is removed due to the introduction of BC (Figure S1b, Supporting Information). During the oxidative polymerization, the pre‐polymerization solution rapidly changed from white to brown within 30 s (Figure S2, Supporting Information), indicating that polydopamine began to form. Dopamine groups reacted with the amino groups on the Gel through Schiff base and Michael addition reactions to form the crosslinked structure.^[^ ^26^ ^]^ At the same time, BC was also oxidized into the oxidized BC with aldehyde groups,^[^ ^27^ ^]^ which could crosslink with dopamine groups^[^ ^28^ ^]^ and Gel's amino groups^[^ ^27^ ^]^ by Schiff base reactions (Figure S3, Supporting Information). Finally, after multiple washing with deionized water and freeze‐drying, the dried BGP (DBGP) cryogel product was obtained (Figure 1d). DBGP cryogel can be personalized with different molds to meet the needs of different shapes and depths of wounds (**Figure** **2**a).

As shown in Fourier transform infrared (FTIR) spectra of DA, BC, Gel, and DBGP cryogel (Figure 2b), the absorption peaks at 3100–3500 cm^−1^ for DBGP cryogel are attributed to ‐OH and ‐NH_2_ stretching vibrations of BC, Gel and DA.^[^ ^29^ ^]^ Due to the crosslinking of Gel, BC and DA, the typical characteristic absorption peaks of amide I (C = O) and amide II (N‐H) of Gel at 1646 cm^−1^ and 1542 cm^−1^ weaken,^[^ ^29^ ^]^ and the absorption peak of amide I is shifted to 1660 cm^−1^. In addition, there is a new absorption peak at 1623 cm^−1^ for C = N stretching vibration in DBGP cryogel, resulting from the formation of the Schiff base.^[^ ^30^ ^]^ XPS was also performed to characterize DBGP cryogel. As shown in Figure 2c, DBGP cryogel shows a characteristic peak of I 3d_5/2_ around the binding energy of 620 eV, indicating the presence of iodine.^[^ ^31^ ^]^ High resolution I 3d_5/2_ XPS spectrum further confirms the existence of I_2_ and sodium iodides in DBGP cryogel^[^ ^32^ ^]^ (Figure S4, Supporting Information). The I_2_ and sodium iodides could be produced in situ by reduction of sodium periodate during redox reactions and provide antibacterial activity for DBGP cryogel.^[^ ^33^ ^]^ The above results confirm the successful synthesis of DBGP cryogel.

It is well known that wound infection and exudate interact with each other.^[^ ^34^ ^]^ The infection will aggravate the exudation of wound exudate, and exudate, as a natural bacterial culture medium, can further aggravate the infection, forming a vicious circle.^[^ ^34^, ^35^ ^]^ Therefore, the ideal wound dressing requires suitable swelling property and breathability to rapidly absorb exudate, promote exudate evaporation, and facilitate oxygen penetration, which can prevent excessive wound hydration, reduce the risk of infection, and improve the local metabolism of wound tissue to promote effective infected wound healing.^[^ ^36^ ^]^ As shown in scanning electron microscopy (SEM) image of Figure 2d, DBGP cryogel has interpenetrated porous structure with pore sizes in the range of 500–750 µm, which provides good breathability and exudate management performance of dressings. In swelling experiments, DBGP cryogel exhibits ideal absorption capacity for PBS with a swelling ratio of 3800% (Figure 2e), which is higher than most of the reported dressings (Table S2, Supporting Information). The breathability test shows that the water vapor transmission rate of the DBGP cryogel is 2368.27 g m^−2^ day^−1^ at 37 °C, which is comparable to that of gauze (2448.54 g m^−2^ day^−1^) and significantly higher than that of Burn Caring^®^ hydrogel (Figure S5, Supporting Information) and most dressings reported previously (Table S2, Supporting Information). The above results clearly demonstrate that DBGP cryogel has ideal swelling property and breathability.

Considering that the good compression strength facilitates the application of cryogels,^[^ ^37^ ^]^ we optimized the compression strength of the DBGP cryogels by modulating the ratio of reactive monomers. Stress‐strain tests were carried out on DBGP cryogels absorbing deionized water to full saturation at 80% compressive strain (Figure S6, Supporting Information). The compressive strength of the cryogels increases with increasing the concentrations of BC and Gel. At the same time, there is no significant correlation between the content of DA and the compressive strength of cryogels. The compressive strength of DBGP cryogel could reach 25 kPa, exceeding that of the reported EDC/NHS cross‐linked gelatin/polydopamine cryogel.^[^ ^20^ ^]^ We also evaluated the compression stability of DBGP cryogel absorbing deionized water to full saturation. After 50 cycles of compression test, the DBGP cryogel keeps its original shape, and still reaches a compressive strength of 20 kPa (Figure 2f), indicating that the cryogel has good compression elasticity.

In deep and irregularly shaped wounds, dressings with shape memory recovery are uniquely advantageous because they can be delivered to deep wounds in a compressed state via an injector, come into contact with exudate or blood, and rapidly expand in volume to fill abnormal wound boundaries.^[^ ^19^, ^38^ ^]^ As shown in Figure 2g, after compression and decompression, DBGP cryogel maintains the compressed shape. However, when feeding water, the DBGP cryogel rapidly absorbs water and returns to its original shape. Subsequently, when the water of the cryogel is squeezed out, it can reabsorb water and return to its original shape upon touching with water again. In addition, DBGP cryogel (original shape of 11 mm in diameter) could be injected using an injector of approximately 5 mm in diameter after compression, and then absorbs water immediately to restore its original shape (Figure S7, Supporting Information). These results demonstrate that the DBGP cryogel has excellent water‐triggered shape recovery property. When DBGP cryogel is compressed, the interpenetrated porous structure collapses, and the cryogel stays compressed even after the pressure is released; once the compressed cryogel absorbs water, the stored elastic energy is released, and the cryogel regains its original shape and structure^[^ ^17^ ^]^ (Figure S8, Supporting Information). Additionally, due to the polydopamine component, DBGP cryogel could cling more red blood cells and platelets than commercial gelatin sponge (GS) and gauze, and activate the platelets to grow filamentous pseudopods (Figure S9, Supporting Information), which facilitates the hemostatic performance of dressing.^[^ ^20^ ^]^ The above results suggest that DBGP cryogel is capable of filling irregular wounds and has the potential to enhance coagulation as well as hemostasis.

**Figure 3:**
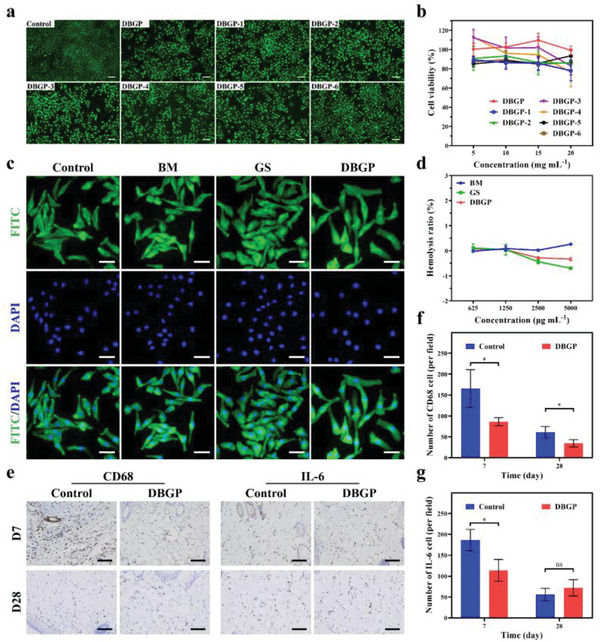
a) Live/dead cell staining images of DBGP cryogels (scale bar: 100 µm); b) cell viability of DBGP cryogels; c) cytoskeleton staining images of BM, GS, and DBGP cryogel (scale bar: 50 µm); d) hemolysis ratios of BM, GS, and DBGP cryogel; e) immunohistochemical staining images of CD68 and IL‐6 on days 7 and 28 (scale bar: 100 µm); f) quantitative analysis of CD68; g) quantitative analysis of IL‐6 (*n* = 3; *p < 0.05, **p < 0.01, ***p < 0.001).

Excellent biocompatibility is a fundamental requirement for biomedical materials. Live/dead cell staining, CCK‐8 kit, and cytoskeleton staining assays were used to evaluate the cytocompatibility of the cryogels. The results of live/dead cell staining experiments show that most of the L929 cells in the cryogel groups are green (green and red cells are live and dead cells, respectively), which is similar to the control group (**Figure** **3**a). The CCK‐8 experiments show that after co‐culturing with various concentrations of cryogel extracts (5, 10, 15, and 20 mg mL^−1^) for 24 h, the cell viability of L929 cells all exceeds 80% and even exceeds 100% in DBGP cryogel groups (Figure 3b). Furthermore, according to the cytoskeleton staining images, the cells in DBGP cryogel group have a spindle‐shaped morphology with intact nuclei and cytoplasm (cytoplasm stains green and nucleus stains blue), consistent with the cellulose membrane (BM), gelatin sponge (GS), and control group (Figure 3c). To further evaluate the hemocompatibility of DBGP cryogel, the hemolysis ratios of BM, GS, and DBGP cryogel dispersion liquids with various concentrations (625, 1250, 2500, and 5000 µg mL^−1^) were tested. The supernatant of all the experimental groups, negative PBS group, and positive Triton X‐100 group was photographed after centrifugation. The cryogel group presents light yellow similar to the negative PBS group, while the positive Triton X‐100 group is bright red (Figure S10, Supporting Information). As shown in Figure 3d, the hemolysis ratios of DBGP cryogel **is** lower than the negative group at all concentrations, demonstrating good hemocompatibility of DBGP cryogel. The histocompatibility of DBGP cryogel was tested by dorsal subcutaneous implantation on rat models for days 7 and 28. According to immunohistochemistry staining analysis, DBGP cryogel exhibits a milder inflammatory response than GS (control) with lower expression of CD68 and IL‐6 (Figure 3e–g), indicating DBGP cryogel has better histocompatibility than GS. Furthermore, a skin irritation assay was also performed on the rat model. The safety of DBGP cryogel was evaluated by the skin irritation test according to the scoring criteria (Table S3, Supporting Information). After 24 and 72 h, no signs of erythema, edema, allergies or any other symptoms are observed on the rats' dorsal skin (Table S4, Figure S11, Supporting Information), indicating that the DBGP cryogel does not cause any skin irritations. These results suggest that DBGP cryogel exhibits good biocompatibility.

**Figure 4:**
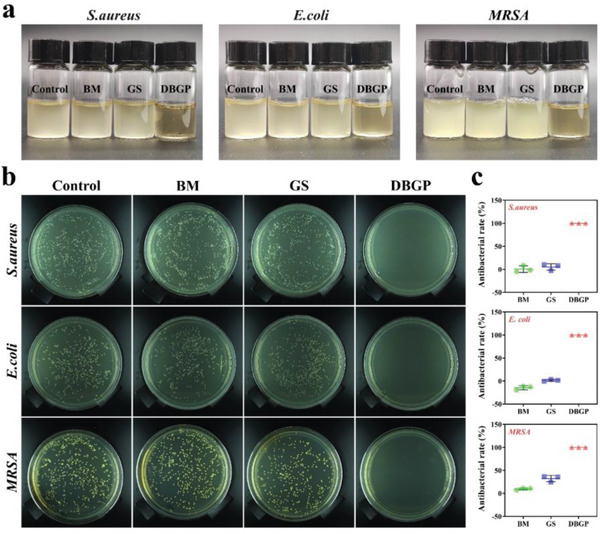
a) Digital photos of *S. aureus*, *E. coli* and *MRSA* suspensions after co‐cultured with BM, GS and DBGP cryogel for 12 h; b) digital photos of *S. aureus*, *E. coli*, and *MRSA* bacterial colonies on agar plates after co‐cultured with BM, GS and DBGP cryogel for 12 h; c) antibacterial rates of BM, GS and DBGP cryogel for *S. aureus*, *E. coli* and *MRSA*.

With the massive use of antibiotics, drug‐resistant bacteria have become a frequent flora on the wound, significantly increasing the difficulty of treating infected diabetic wounds.^[^ ^39^ ^]^ Therefore, diabetic wound dressings need to have excellent antibacterial properties. To evaluate the antibacterial effectiveness of DBGP cryogel that contains antibacterial I_2_ and sodium iodides produced in situ by sodium periodate reduction, *Staphylococcus aureus* (*S. aureus*, a gram‐positive bacteria), *Escherichia coli* (*E. coli*, a gram‐negative bacteria), and Methicillin‐resistant *Staphylococcus aureus* (*MRSA*, a drug‐resistant bacteria) were employed to perform the antibacterial **tests**. Firstly, BM, GS, and DBGP cryogel were co‐cultured with the three mentioned bacteria solutions (10^8^ CFU mL^−1^, 3 mL) for 12 h, respectively. The results show that the culture solution of DBGP cryogel remains clarified, while the rest groups are turbid because of the proliferation of bacteria (**Figure** **4**a). Subsequently, the antibacterial properties of the DBGP cryogel were further evaluated by the agar plate coating experiments (Figure 4b). The DBGP cryogel group shows high antibacterial rates of about 100% against *S. aureus*, *E. coli* and *MRSA*, while there exist massive bacteria on the agar plates of BM, GS, and control groups (Figure 4c). The bacterial morphology on the surfaces of BM, GS, and DBGP was observed by SEM (Figure S12, Supporting Information). The bacterial morphology in the BM and GS groups does not change. In sharp contrast, the bacterial morphology in the DBGP cryogel group exhibits apparent depression and deformation, indicating their death. The above results demonstrate that DBGP cryogel has excellent broad‐spectrum antibacterial properties. Compared with most of reported cryogels,^[^ ^18^, ^19^, ^38^ ^]^ which only exhibited antibacterial ability for *S. aureus* and *E. coli*, our DBGP cryogel could be more suitable for the clinical needs of infected diabetic wounds.

**Figure 5:**
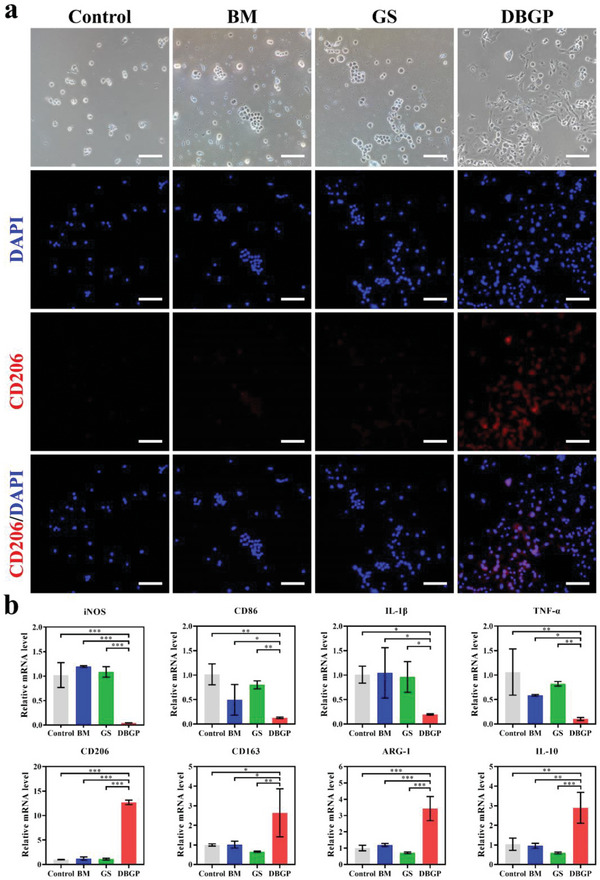
a) Morphological and immunofluorescence staining (DAPI and CD206) images of M1 macrophages (control) and M1 macrophages after co‐cultured with BM, GS, and DBGP cryogel (scale bars: 100 µm); b) relative mRNA expression levels of macrophages after co‐cultured with BM, GS, and DBGP cryogel for iNOS, CD86, IL‐1β, TNF‐α, CD206, CD163, ARG‐1, and IL‐10 (*n* = 3; *p < 0.05, **p < 0.01, ***p < 0.001).

The microenvironment of diabetic wound is not conducive to the transformation of macrophages from M1 (pro‐inflammatory) to M2 (anti‐inflammatory) phenotype, leading to chronic inflammation of the wound and prolonged healing time.^[^ ^40^ ^]^ The ability of DBGP cryogel to induce macrophage polarization from M1 to M2 phenotype was investigated in vitro. The lipopolysaccharide (LPS) induced M1 phenotype served as control group. As shown in **Figure** **5**a, the majority of macrophages in the BM and GS groups show a similar morphology to the control group, with a rounded shape and short pseudopodia, which are typical M1 macrophages. While the macrophages in the DBGP cryogel group exhibit the typical characteristics of M2 macrophages with a spindle shape and long pseudopodia^[^ ^40^ ^]^ (Figure 5a). Immunofluorescence staining was performed on the M2 macrophage surface marker CD206 to further assess the polarization status of macrophages. Macrophages co‐cultured with DBGP cryogel show much more significant CD206 staining compared with control, BM and GS groups (Figure 5a). In addition, the qRT‐PCR test results show that DBGP cryogel has the highest mRNA expression levels of M2 macrophage marker genes (including CD206, CD163, ARG‐1, and IL‐10) compared to the other groups (Figure 5b). Meanwhile, the opposite trend is observed in M1 macrophage marker genes (including iNOS, CD86, IL‐1β, and TNF‐α) with significantly decreased expression levels (Figure 5b). All these results suggest that DBGP cryogel can effectively induce polarization of macrophages from M1 to M2 phenotype.

**Figure 6:**
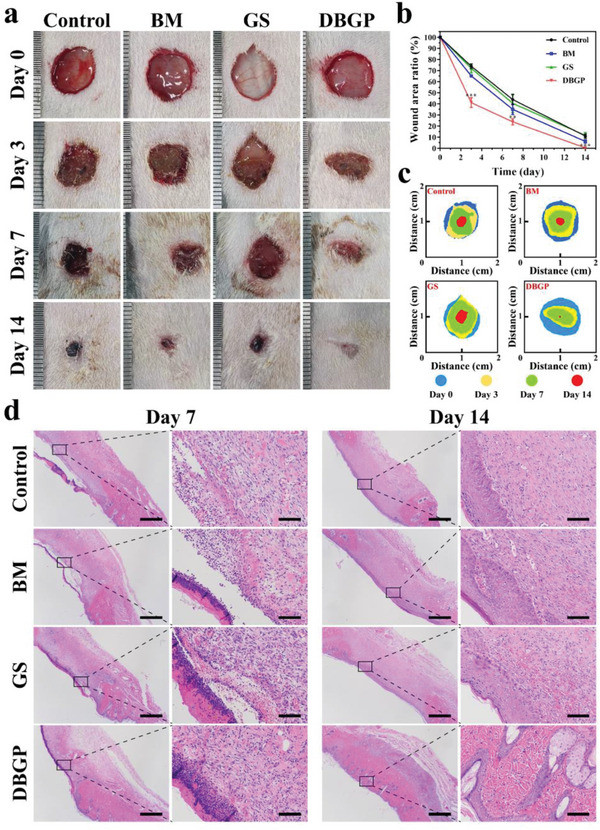
a) Digital photos of wounds in the control, BM, GS, and DBGP cryogel groups on days 0, 3, 7 and 14; b) wound area ratios at different days for each group (*n* = 3; *p < 0.05, **p < 0.01, ***p < 0.001); c) wound traces at different periods; d) H&E staining images of wound tissues on days 7 and 14 (scale bars: 1000 µm for column 1 and 3; 100 µm for column 2 and 4).

A full‐thickness cutaneous wound model of type I diabetic rats infected with *S. aureus* was established to assess the wound healing of gauze (control), BM, GS, and DBGP cryogel. On day 1, the pus occurs in the wounds, indicating the successful establishment of the infected wound model (Figure S13, Supporting Information). The wound area of different groups was observed and calculated on days 3, 7, and 14. The wound area of the DBGP cryogel group (41.07 ± 3.57%) is significantly reduced at the early healing stage on day 3 compared with the control group (73.86 ± 1.63%), and further reduced (23.75 ± 2.24%) on day 7 (**Figure** **6**a, b). On day 14, the wound in the DBGP cryogel group is healed and covered with new epithelium, while the other groups still have about 6%−12% of the open wound (GS group: 11.51 ± 2.11%, BM group: 6.14 ± 1.19%, control group: 11.04 ± 1.49%) (Figure 6a,b). The wound healing schematic further visualizes the change of the wounds area (Figure 6c). H&E staining of wound tissues shows that all groups exhibit obvious wounds with few granulation tissues on day 7 (Figure 6d). However, on day 14, the DBGP cryogel group has the shortest wound length, and significantly more collagenous fibers than the control, BM, and GS groups (Figure 6d).

**Figure 7:**
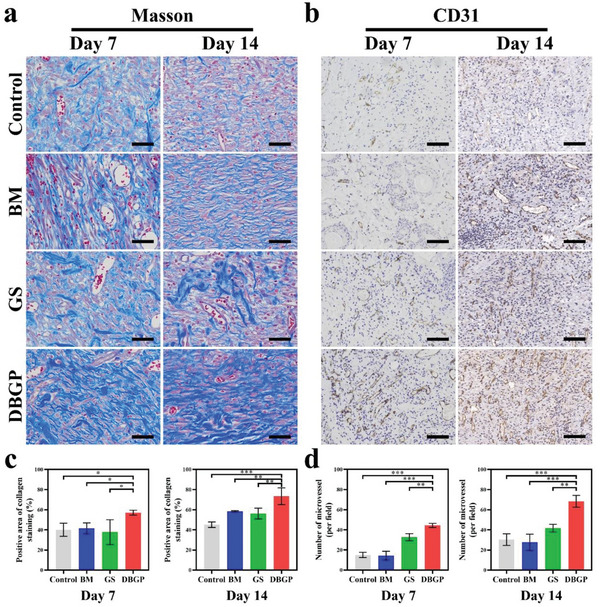
a) Masson staining images and b) CD31 immunohistochemical staining on days 7 and 14 (scale bars: 50 µm for Figure 7a and 100 µm for Figure 7b); c) quantitative analysis of collagen density and d) the microvessels on days 7 and 14 (*n* = 3; *p < 0.05, **p < 0.01, ***p < 0.001).

Proper collagen deposition and remodeling are critical during the remodeling phase to promote tissue tensile strength and healing.^[^ ^12^ ^]^ The collagen deposition and remodeling of the regenerated tissue on days 7 and 14 were evaluated by Masson staining (**Figure** **7**a). According to the results, the DBGP cryogel group shows the highest collagen density, reaching 57.2% and 73.5% on days 7 and 14, respectively, followed by the BM (41.5% and 58.4%), GS (37.7% and 56.2%), and control (40.1% and 45.2%) groups (Figure 7c). Neovascularization is required for chronic wound healing because it provides nutrition to healing‐associated cells and supports the formation of new granulation tissue.^[^ ^41^ ^]^ The newly formed microvessels are visualized and counted at the wound site by CD31 staining (Figure 7b). The results show that the level of microvessels in the DBGP cryogel group is significantly higher than the other groups on both days 7 and 14. Specifically, on day 7, the number of microvessels is 15, 14, 33, and 44/HP for the control, BM, GS, and DBGP cryogel groups, respectively, while it is 30, 28, 42, and 68/HP on day 14 (Figure 7d). These results suggest that DBGP cryogel can enhance collagen deposition and angiogenesis in infected diabetic wounds and promotes healing.

**Figure 8:**
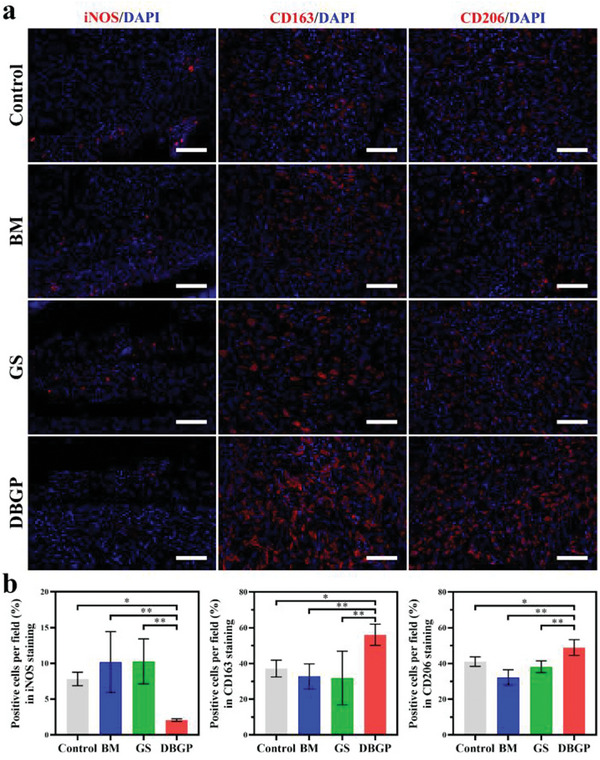
a) Immunofluorescence staining images of macrophage markers iNOS, CD163, and CD206 from wound tissue (scale bar: 100 µm); b) quantitative analysis of iNOS, CD163, and CD206 (*n* = 3; *p < 0.05, **p < 0.01, ***p < 0.001).

The contribution of DBGP cryogel for macrophage polarization was further investigated in the infected diabetic wounds. Immunofluorescence staining for iNOS (surface marker of M1 macrophages), CD163 and CD206 (surface markers of M2 macrophages) were performed on wound tissue on day 7 to assess the distribution of M1 and M2 macrophages (**Figure** **8**a). The iNOS positivity levels in the control, BM, and GS groups (7.8%, 10.2%, and 10.3%) are significantly higher than that of the DBGP cryogel group (2.1%) (Figure 8b). In contrast, the DBGP cryogel group has the highest positive expression of CD163 and CD206, reaching 56% and 49%, respectively, which is superior to that of BM (32.9% and 32.2%) and GS (31.9% and 38.1%) (Figure 8b). The above results demonstrate that DBGP cryogel can induce macrophage polarization to the M2 phenotype during infected diabetic wound healing, facilitating the transition from the inflammatory phase to the proliferative and healing phase of the wound.

## 3 Conclusion

In this study, a novel class of porous DBGP cryogel dressing has been prepared with BC, Gel, and DA by the facile one‐pot method in the presence of sodium periodate. Remarkably, sodium periodate is an oxidizing agent for the reaction system, and its reduction I_2_ and sodium iodides impart good antibacterial properties to the cryogels, and the BC improves the cross‐linking and mechanical properties of the cryogels. The as‐obtained DBGP cryogel exhibits good biocompatibility, breathability, and exudate management properties and can efficiently induce the polarization of macrophages from M1 to M2 phenotype. Our DBGP cryogel demonstrates faster tissue regeneration, enhanced collagen deposition, and neovascularization when treating the infected diabetic wounds. We hope that DBGP cryogel could become a high‐performance dressing for infected diabetic wounds with great clinical application potential.

## 4 Experimental Section

All methods can be found in the accompanying supplementary information.

## Conflict of Interest

The authors declare no conflict of interest.

## Supporting information

Supporting Information

## Data Availability

Research data are not shared.
