# Supplementary material for: Biocompatible Cryogel with Good Breathability, Exudate Management, Antibacterial and Immunomodulatory Properties for Infected Diabetic Wound Healing
Source: Adv Sci (Weinh). 2023 Sep 4;10(31):2304243. doi: 10.1002/advs.202304243 (PMC10625128; doi:10.1002/advs.202304243)
Supplement: Supplementary file 1 — Supporting Information [file ADVS-10-2304243-s001.pdf]

ADVANCED SCIENCE
Open Access

Supporting Information

for *Adv. Sci.*, DOI 10.1002/advs.202304243

Biocompatible Cryogel with Good Breathability, Exudate Management, Antibacterial and Immunomodulatory Properties for Infected Diabetic Wound Healing

Yang Li, Zifeng Yang, Qi Sun, Ruijun Xu, Renjie Li, Dingcai Wu, Rongkang Huang*, Feng Wang* and Yong Li**

Supporting Information

Biocompatible Cryogel with Good Breathability, Exudate Management, Antibacterial and Immunomodulatory Properties for Infected Diabetic Wound Healing

Yang Li, Zifeng Yang, Qi Sun, Ruijun Xu, Renjie Li, Dingcai Wu*, Rongkang Huang*, Feng Wang*, Yong Li*

Y. Li, Q. Sun, R. Xu, Prof. Y. Li

School of Medicine

South China University of Technology

Guangzhou, 510006, China

E-mail: liyong@gdph.org.cn

Y. Li, Dr. Z. Yang, Q. Sun, R. Xu, Dr. R. Li, Dr. F. Wang, Prof. Y. Li

Department of Gastrointestinal Surgery, Department of General Surgery

Guangdong Provincial People's Hospital (Guangdong Academy of Medical Sciences)

Southern Medical University

Guangzhou, 510080, China

E-mail: wangfeng@gdph.org.cn

Dr. R. Li, Dr. F. Wang, Prof. Y. Li

Guangdong Cardiovascular Institute

Guangdong Provincial People's Hospital

Guangdong Academy of Medical Sciences

Guangzhou, 510080, China

Prof. D. Wu

PCFM Lab

School of Chemistry

Sun Yat-sen University

Guangzhou 510006, China

E-mail: wudc@mail.sysu.edu.cn

Dr. R. Huang

Department of General Surgery (Colorectal Surgery)

Guangdong Institute of Gastroenterology

Biomedical Innovation Center

Guangdong Provincial Key Laboratory of Colorectal and Pelvic Floor Diseases

The Sixth Affiliated Hospital

Sun Yat-sen University

Guangzhou 510655, China

E-mail: huangrk3@mail.sysu.edu.cn

Materials and Method

1. Materials

Bacterial cellulose (BC) aqueous dispersion (0.65 wt%) was purchased from Guilin Qihong Technology Co., Ltd. (China). Gelatin (Gel, type B, from bovine skin) was purchased from Shanghai Yuanye Bio-Technology Co., Ltd. (China). Dopamine hydrochloride (DA) and sodium periodate (SP) were purchased from Shanghai Macklin Biochemical Technology Co., Ltd. (China). The commercial gauze was purchased from Cofoe Medical Technology Co., Ltd. (China), and the commercial gelatin sponge was purchased from Jiangxi Xiangen Medical Technology Development Co., Ltd. (China). Burn Caring® hydrogel dressing (HD-L type) was purchased from Changchun JA Biotech Co., Ltd. (China). Fetal bovine serum (FBS), Dulbecco's Modified Eagle Medium (DMEM), and penicillin-streptomycin solution were purchased from Gibco BRL (USA). The cell counting kit-8 (CCK-8) was purchased from Labgic Technology Co., Ltd. (China). The live/dead cell viability kit (Calcein-AM/PI) was purchased from Shanghai Xinyu Biotechnology Co., Ltd. (China). The Actin-Tracker Green-488 was purchased from Beyotime Biotech Inc. (China). The rabbit blood and platelet-rich plasma (PRP) were purchased from Guangzhou Hongquan Biotechnology Co., Ltd. (China). RNA-Quick purification kit was purchased from Shanghai Yishan Biotechnology Co., Ltd. (China). PrimeScript™ RT Master Mix (Perfect Real Time) and TB Green® Premix Ex Taq™ (Tli RNaseH Plus) were purchased from Takara Biomedical Technology (Beijing) Co., Ltd. (China). *Staphylococcus aureus* (*S. aureus*, ATCC 25923), *Escherichia coli* (*E. coli*, ATCC 25922), and methicillin-resistant *Staphylococcus aureus* (*MRSA*, ATCC 43300) were obtained from the department of laboratory medicine, Guangdong Provincial People's Hospital (China). RAW264.7 macrophages and mouse fibroblast L929 cells were purchased from Jiangsu Keygen Biotech Co., Ltd. (China). All other chemicals were purchased from Shanghai Macklin Biochemical Technology Co., Ltd. (China). All solvents and chemicals were purchased from commercial sources and used as received, unless otherwise noted. The male

Sprague Dawley (SD) rats (12-week-old, 200-250 g) were purchased from laboratory animal center of Sun Yat-sen University (China). Histological analysis was tested by Wuhan Servicebio Technology Co., Ltd. (China).

2. Synthesis of GP cryogel

The GP cryogel was synthesized using the following procedure. Firstly, 10 mL Gel aqueous solution (4%) was added to a beaker and stirred. 0.3 mL DA aqueous solution (18%) was added to the beaker and thoroughly mixed. Subsequently, 0.6 mL SP aqueous solution (6%) was added to the mixture and stirred for 30 s. After that, the prepared cryogel precursor solution was transferred to mold, placed in a freezer at -20 °C for 24 h, and thawed at room temperature to form the GP cryogel.

3. Synthesis of DBGP cryogels

The dried bacterial cellulose/gelatin/polydopamine (DBGP) cryogels were synthesized using a one-pot method reaction. Firstly, 10 mL BC aqueous dispersion and 10 mL Gel aqueous solution were added to a beaker and stirred. Next, DA aqueous solution (18%) was added to the beaker and thoroughly mixed. After that, SP aqueous solution (6%) was added to the mixture and stirred for 30 s. The color of the solution gradually changed from white to brown. Subsequently, the prepared cryogel precursor solution was transferred to mold, placed in a freezer at -20 °C for 24 h, and then thawed at room temperature to form BGP cryogels. After washing with deionized water for 7 days (displace the water every 12 h) to remove the unreacted monomers as well as freeze-drying for 2 days, the DBGP cryogels were obtained. The preparation parameters of DBGP cryogels are listed in Table S1.

4. Microstructure characterization

The FT-IR spectra of DA, BC, Gel, and DBGP cryogel were tested using a FT-IR spectrometer (Nexus 670, Thermo Nicolet) in the range of 4000-500 cm^{-1}. The XPS spectrum of DBGP cryogel was recorded by X-ray photoelectron spectrometer (Nexsa, Thermo Fisher Scientific). Morphologies of the DBGP cryogel in its original state, compressed state, and recovered state were observed using a field emission scanning

electron microscope (S-4800, Hitachi). Before the observation, all materials were sprayed with gold.

5. Compressive tests of DBGP cryogels

Compressive tests of the DBGP cryogels were performed on a universal mechanical testing machine (WD-5A, Guangzhou Experimental Instrument Factory) at room temperature. The tested cryogels were prepared in a cylindrical shape with 12 mm in height and 20 mm in diameter. Before the test, the cryogels were saturated with deionized water to full saturation. The stress-strain curves were obtained by compressing the cryogels at a maximum compression strain of 80% with a strain rate of 100 μm s^{-1}. The cyclic compression stress-strain curves of the DBGP cryogel were obtained by compressing the cryogel to 80% strain and then releasing it to 0% strain for 50 cycles. The compression and release rates were set at 100 μm s^{-1}.

6. Swelling ratio test of DBGP cryogels

The DBGP cryogels were first weighed and recorded as W_0, then immersed in PBS buffer (pH 7.4) at room temperature for 48 h. Finally, the samples were taken out and their weight was recorded as W_1. The swelling ratio (SR) was calculated according to Eq. (1):

$$SR = \frac{W_1 - W_0}{W_0} \times 100\% \qquad (1)$$

7. Injectability of DBGP cryogel

The DBGP cryogel with a diameter of 11 mm was compressed and loaded into an injector with an inner diameter of 5 mm. The compressed cryogel was then injected into the water to restore its shape.

8. Breathability of DBGP cryogel

To measure the breathability of DBGP cryogel, a centrifuge tube containing 20 mL deionized water was covered with DBGP cryogel. The area of the tube mouth was recorded as A (m^2). Another two identical centrifuge tubes were covered with gauze and hydrogel as the controls. The borders of the tubes were sealed to prevent the loss of water vapor at the boundaries, and their total weight was recorded as W_0. The

centrifuge tubes were then placed in a constant temperature oven at 37 °C for 1 day, and their total weight was recorded as W_1. Three parallel tests were conducted for each sample. The water vapor transmission rate (WVTR, g m^{-2} day^{-1}) was determined by measuring the mass loss of water in the centrifuge tubes according to Eq. (2):

$$WVTR = \frac{W_0 - W_1}{A} \qquad (2)$$

9. Biocompatibility of DBGP cryogels

Cell cytotoxicity and proliferation were measured using the cell counting kit-8 reagent (CCK-8). Firstly, the cryogels were sterilized using UV irradiation for 1 h and then immersed in complete medium for 24 h to obtain cryogel extracts with the concentrations of 5, 10, 15, and 20 mg mL^{-1}. L929 fibroblasts at a density of 10^5 mL^{-1} were cultured in 96-well plates with 100 μL DMEM, supplemented with 10% fetal bovine serum and 1% penicillin-streptomycin, and then incubated in a cell culture incubator (37 °C, 5% CO_2) for 24 h. After the L929 fibroblasts adhered to the plate, the medium was replaced with different concentrations of cryogel extracts and incubated for 24 h. Subsequently, 10 μL CCK-8 was added to each well and incubated for 4 h. Cells seeded into the culture without cryogel extracts served as the control group. Finally, the obtained supernatants were transferred to another 96-well plate, and the optical density (OD) was measured at 450 nm using a full-wavelength microplate reader (Multiskan GO, Thermo Fisher Scientific) to evaluate cell viability.

The live/dead staining process was as follows: 500 μL L929 fibroblast suspension (10^5 mL^{-1}) was inoculated into 24-well plates and cultured in a 5% CO_2 atmosphere at 37 °C for 24 h. The medium was replaced with DBGP cryogel extract (10 mg mL^{-1}) and incubated for 24 h. Subsequently, the cells were stained with the live/dead cell detection kit and visualized using an inverted fluorescent microscope (TI-S, Nikon). Cells seeded into the culture without cryogel extracts served as the control group.

The cytoskeleton staining process was described as follows: 500 μL L929 fibroblast suspension (10^5 mL^{-1}) was inoculated into 24-well plates and cultured in a 5% CO_2 atmosphere at 37 °C for 24 h. Sterilized BM, GS, and DBGP cryogel (10 mg) were

added to the plates and incubated for 24 h. Afterwards, the medium and samples were removed, and the cells were washed 3 times with PBS. The cells were fixed with 4% paraformaldehyde, washed 3 times with PBS, permeabilized and closed with 0.1% Triton X-100/1% NBS-PBS for 20 min. The cells were then incubated with Actin-Tracker Green at room temperature and in the dark for 1 h to visualize the cytoskeleton. After that, the cells were washed 3 times with 0.1% Triton X-100/PBS and stained with DAPI in the dark to visualize the cell nucleus. An inverted fluorescence microscope (TI-S, Nikon) was used to identify the nucleus and cytoskeleton (495 nm for Actin-Tracker Green excitation and 364 nm for DAPI excitation).

Histocompatibility testing was performed by subcutaneously implanting cryogels in the dorsal side of rats. First, male Sprague Dawley rats (200-250 g) were anesthetized, and a surgical incision was made symmetrically on the left and right sides of the back spine of each rat. The GS and DBGP cryogel (diameter: 10 mm, height: 2 mm) were implanted into the subcutaneous incisions. Rats were euthanized on days 7 and 28 after surgery. Subcutaneous tissues were excised and fixed for immunohistochemical staining of CD68 and IL-6 to assess acute and chronic inflammatory responses of the materials.

The skin irritation test of DBGP cryogel was performed on rats. Rat skin was prepared and naturally recovered with materials for 24 h. Before the experiment, the skin on the back of the rat was washed with normal saline. DBGP cryogel was applied to the right side, while GS was applied to the left side as the control. The skin of the test area was photographed after 24 and 72 h to check for erythema, oedema, allergy, and other symptoms.

10. Red blood cell and platelet adhesion of DBGP cryogel

Gauze, GS, and DBGP cryogel with 5 mm height and 10 mm diameter were soaked in PBS at 37 °C for 1 h and then removed. Afterwards, rabbit blood was dropped onto the sample surface and incubated at 37 °C for 5 min. Similarly, platelet-rich plasma (PRP) was dropped onto the surface of another set of samples and incubated at 37 °C for 1 h.

All samples were then washed 3 times with PBS to remove physically attached red blood cells and platelets. After that, the samples were fixed with 2.5% glutaraldehyde for 1 h and dehydrated in 50%, 60%, 70%, 80%, 90%, and 100% ethanol solutions with 10-minute intervals. Finally, the samples were freeze-dried to observe the adhesion of platelets and erythrocytes on the sample surfaces using SEM (Sigma 300, Zeiss). All materials were gold sprayed prior to observation.

11. Hemocompatibility of DBGP cryogel

Rabbit blood was centrifuged at 1300 rpm for 10 min to obtain the erythrocytes. The erythrocytes were washed 3 times and diluted to 5% (v/v) with PBS. The BM, GS, and DBGP cryogel were then immersed in PBS and homogenized using a tissue grinder to prepare dispersions of different concentrations (625, 1250, 2500, and 5000 μg mL^{-1}). The dispersion liquid (500 μL) and erythrocyte suspension (500 μL) were added to a 2 mL tube, gently mixed, incubated at 37 °C for 1 h, and centrifuged at 1300 rpm for 10 min. The supernatant (500 μL) was then transferred separately to new tubes and centrifuged at 13,000 rpm for another 10 min to remove sample particles thoroughly. The as-obtained supernatant (100 μL) was added to a 96-well plate to measure the absorbance at 540 nm using a full wavelength microplate reader. 0.1% Triton X-100 was used as the positive control and PBS was used as the negative control. The hemolysis ratio was calculated according to Eq. (3):

$$Hemolysis\ ratio = \frac{A_p - A_b}{A_t - A_b} \times 100\% \qquad (3)$$

where A_p, A_t, and A_b are the absorbance values of the supernatants from the sample group, Triton X-100 group and PBS groups, respectively. Each group contains three replicates.

12. Antibacterial properties of DBGP cryogel

The BM, GS, and DBGP cryogel (30 mg) were sterilized by UV irradiation for 1 h and added to small glass bottles containing *S. aureus*, *E. coli*, and *MRSA* suspensions (3 mL, 10^8 CFU mL^{-1}). The small glass bottles were then incubated at 37 °C for 12 h. Bacteria-

only suspension was used as the control group and photographed to record the turbidity of the bacterial suspensions.

To further assess the antibacterial abilities, the plate counting method was used, which involved determining the number of colony-forming units (CFU) on agar plates. Firstly, the sterilized BM, GS, and DBGP cryogel (diameter 10 mm, height 2 mm) were co-cultured with 300 μL bacterial suspension (10^7 CFU mL^{-1}) in a 48-well plate at 37 ^{o}C for 12 h. In this assay, a bacterial solution without any treatment was used as the control group. Subsequently, 20 μL of the co-culture solution was pipetted and diluted 10^6 times with PBS, and 20 μL of the as-obtained dilution solution was coated onto agar plates and incubated for 12 h for *S. aureus* and *E. coli* as well as 24 h for *MRSA*. The CFU was measured using ImageJ, and the antibacterial rate (AR) was calculated according to Eq (4):

$$AR = \frac{CFU\ of\ control - CFU\ of\ sample}{CFU\ of\ control} \times 100\% \qquad (4)$$

The samples were then fixed with 2.5% glutaraldehyde for 1 h, followed by dehydration in ethanol solutions of different concentrations (50%, 60%, 70%, 80%, 90%, and 100%) with 10-minute intervals. Afterwards, the samples were freeze-dried, and the morphology of the bacteria on the sample surface was observed using SEM (Sigma 300, Zeiss) after spraying with gold.

13. Macrophage polarization properties of DBGP cryogel in vitro

Briefly, the Raw264.7 macrophages (M0) were induced to M1 macrophages by adding LPS, and then co-cultured with BM, GS, or DBGP cryogel. The macrophage morphology and immunofluorescence staining for macrophage-related markers were observed. The experimental process was as follows: Firstly, 2 mL Raw264.7 macrophages were inoculated into 6-well plates at a density of 10^5 mL^{-1} and co-cultured with the complete medium for 24 h. Subsequently, the medium was changed and LPS (100 ng mL^{-1}) was added to induce Raw264.7 macrophages to M1 macrophages, followed by co-culturing for another 24 h. Afterwards, the medium was changed again, and different samples were added to the 6-well plates and incubated for 48 h. The

macrophages were fixed with 4% paraformaldehyde for 20 min at room temperature, permeabilized with 0.1% (v/v) Triton-X100/PBS for 5 min, and blocked with 1% BSA-PBS for 1 h. The macrophages were then incubated with rabbit polyclonal antibody to CD206 (1:150, Servicebio, China) at 4 °C in the dark, followed by incubation for 1 h with Cy3-labeled goat anti-rabbit IgG secondary antibody (1:200, Servicebio, China) in the dark. Finally, DAPI was added to stain the nuclei. Inverted fluorescence microscopy (TI-S, Nikon) was used to observe the morphology and immunofluorescence of the macrophages.

The qRT-PCR experiment was also performed to evaluate the expression levels of M1 (iNOS, CD86, IL-1β, and TNF-α) and M2 macrophage-associated marker genes (CD206, CD163, ARG-1, and IL-10). Firstly, BM, GS, and DBGP were co-cultured with M1 macrophages for 48 h. Total RNA was then extracted using the RNA Rapid Extraction Kit (RN001, ES Science) and quantified using a spectrophotometer (NanoDrop2000, Thermo Scientific). Next, reverse transcription was performed using the PrimeScript™ RT Master Mix (Perfect Real Time) (RR036A, Takara) kit and a PCR instrument (TAavanced Twin48G, Biometra). The PCR amplification reaction of cDNA was performed using TB Green® Premix Ex Taq™ (Tli RNaseH Plus) (RR820A, Takara) and a real-time fluorescence quantitative PCR instrument (qTOWER³G, Analytik-Jena), and the target gene was accurately quantified by detecting the intensity of the fluorescence signal in the PCR reaction solution. Primers for iNOS, CD86, IL-1β, TNF-α, CD206, CD163, ARG-1, and IL-10 are listed in Table S5. Relative mRNA expression levels of target genes were normalized to control samples and calculated using the $2^{-\Delta\Delta Ct}$ method.

14. In vivo infected diabetic wounds healing assessment

Diabetic model was induced in male SD rats weighing 200-250 g by single intraperitoneal injection of streptozotocin (STZ, 60 mg kg^{-1}) diluted in citrate buffer (pH 4.5). After 1 week, SD rats showed weight loss, urine increase, and blood glucose levels ≥ 16.7 mmol L^{-1}, defined as successful establishment of the type I diabetic model.

[1] Next, diabetic rats were anesthetized and disinfected with a 75% ethanol solution. Four circular skin wounds (10 mm in diameter) were then made on the back of the rats using a skin biopsy punch, and 100 μL *S.aureus* (10^8 CFU mL^{-1}) was added dropwise to form a rat infected diabetic wound model.[2] After 24 h, gauze (control), BM, GS, and DBGP cryogel were covered on the infected wounds and secured with 3M Tegaderm™ film. On days 0, 3, 7, and 14, the healing of the wounds was photographed and recorded. The wound area ratio was measured using ImageJ and calculated according to Eq. (5):

$$Wound\ area\ ratio = \frac{A_{(3,7,14)}}{A_0} \times 100\% \quad (5)$$

where A_0 represents the initial area of wound, and $A_{(3,7,14)}$ represents the area of wound on the 3rd, 7th, or 14th day.

Wound tissues were collected on days 7 and 14 to assess wound healing by H&E, Masson, and CD31 immunohistochemical staining. In addition, the expression levels of iNOS, CD163, and CD206 in the wound tissue on day 7 were examined by immunofluorescence staining to determine the type of macrophages in the wound.

15. Statistical analysis

Statistical analysis was performed using SPSS software (version 26.0 for Windows; SPSS, Chicago, IL, USA). Data was expressed as mean ± standard deviation (SD). Statistical differences between groups were determined by Student's t-test or one-way analysis of variance (ANOVA). Significance levels are indicated as $^*p < 0.05$, $^{**}p < 0.01$, and $^{***}p < 0.001$.

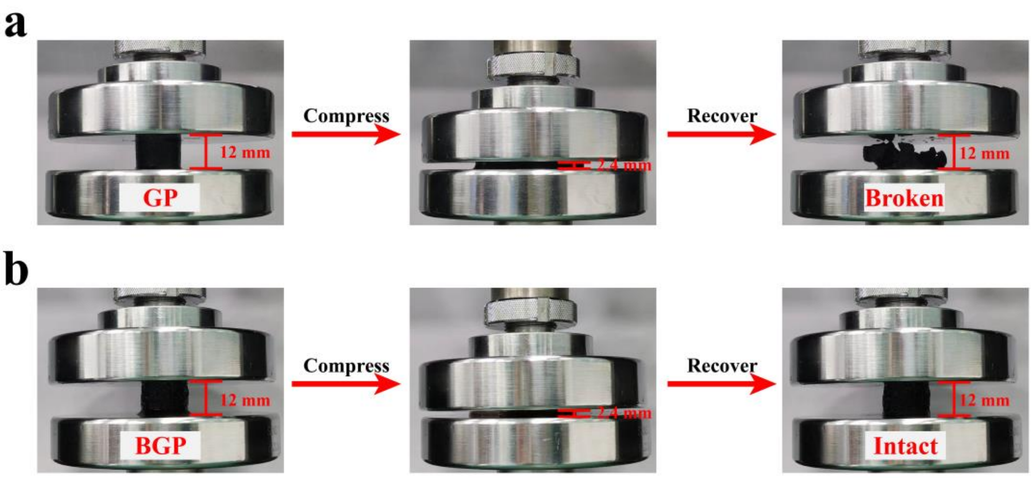

Figure S1. Digital photos of stress compression test of (a) GP and (b) BGP cryogels.

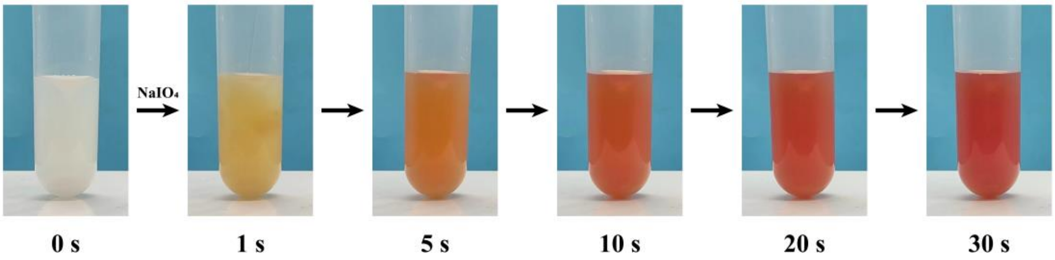

Figure S2. Color change of the BGP cryogel precursor solution with different reaction times.

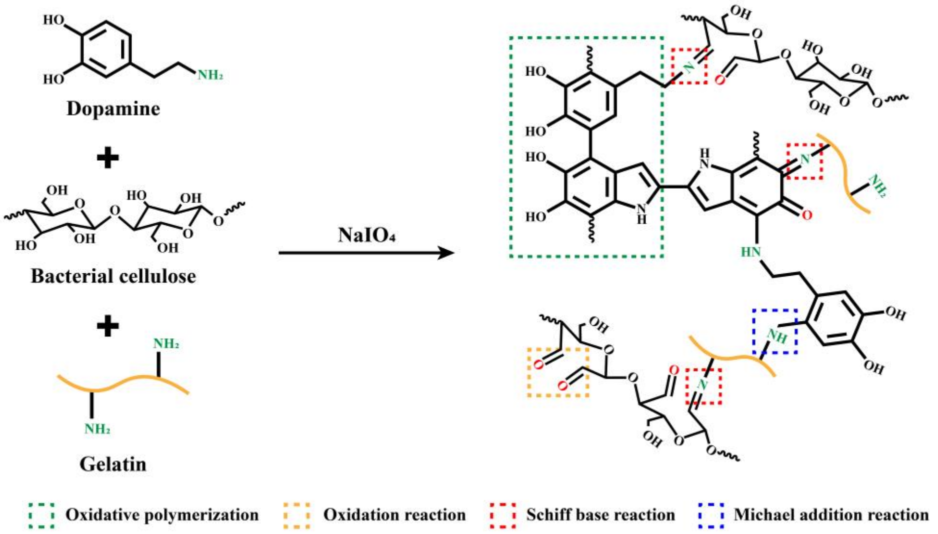

Figure S3. Schematic of main chemical reactions involved in the preparation of BGP cryogel, including oxidative polymerization, oxidation reaction, Schiff base reaction, and Michael addition reaction.

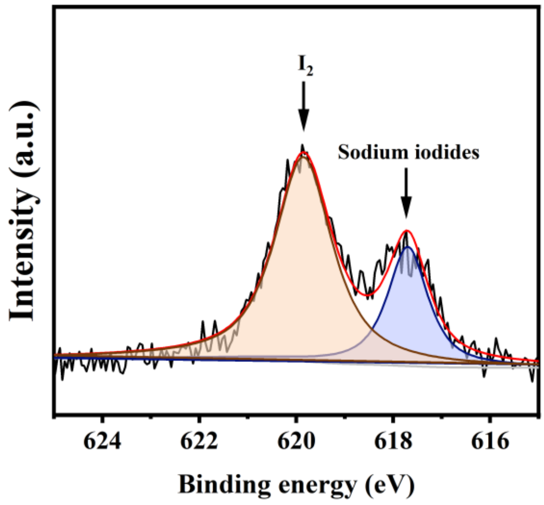

Figure S4. I $3d_{5/2}$ XPS spectrum of DBGP cryogel.

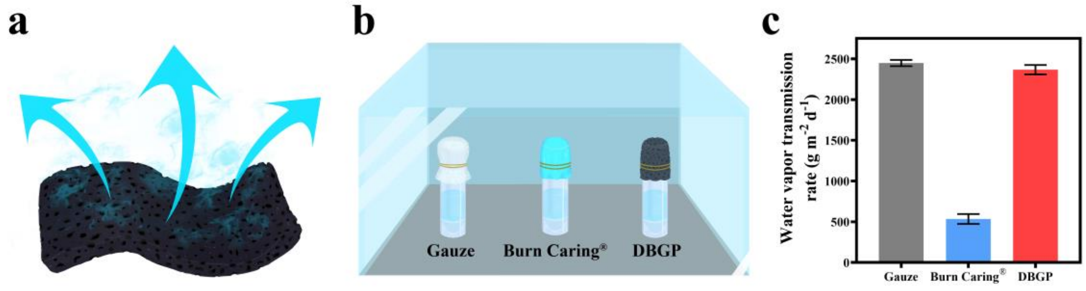

Figure S5. (a) Schematic of the breathability of DBGP cryogel; (b) schematic of the breathability of Gauze, Burn Caring® hydrogel, and DBGP cryogel; (c) water vapor transmissions of Gauze, Burn Caring® hydrogel, and DBGP cryogel.

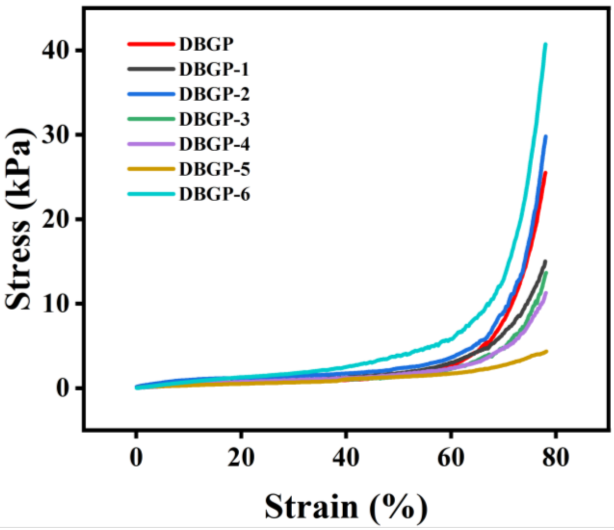

Figure S6. Compressive stress-strain curves of various cryogels.

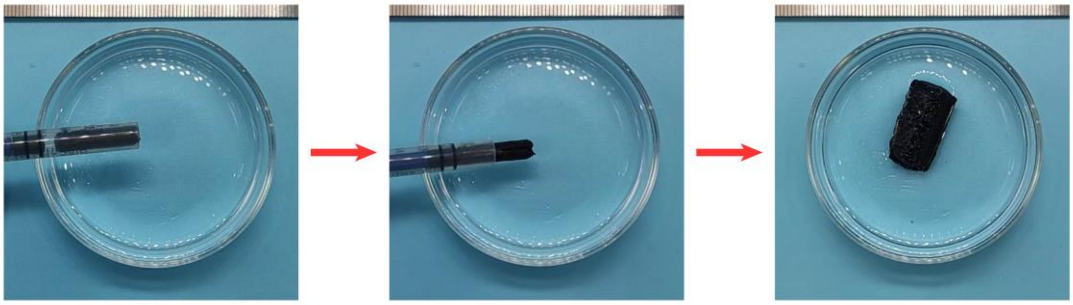

Figure S7. Digital photos of injection process of DBGP cryogel.

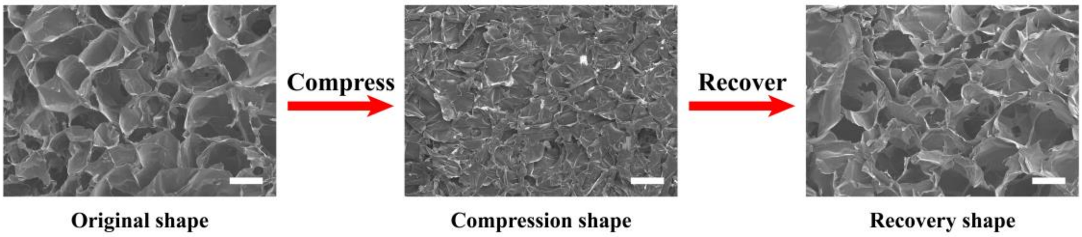

Figure S8. SEM images of DBGP cryogel in original shape, compression shape, and recovery shape. Scale bar: 500 μm.

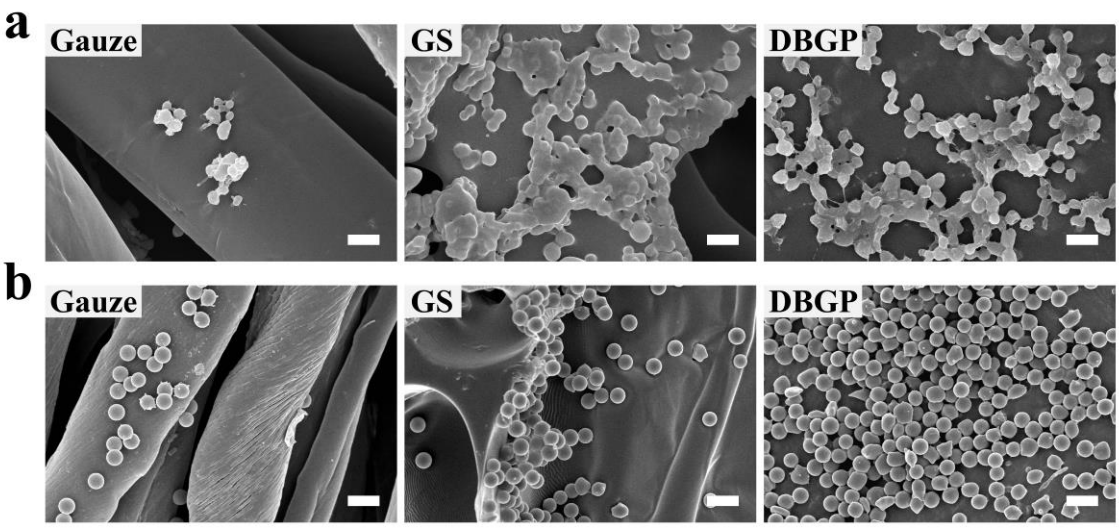

Figure S9. (a) SEM images of platelets adhering to gauze, GS, and DBGP cryogel. Scale bar: 2 μm. (b) SEM images of red blood cells adhering to gauze, GS, and DBGP cryogel. Scale bar: 5 μm.

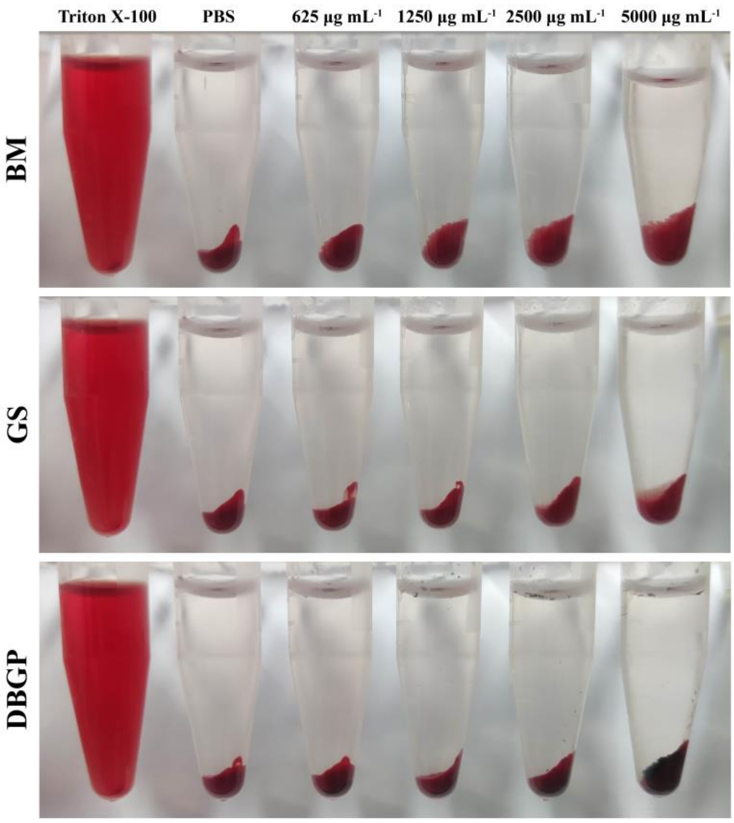

Figure S10. Digital photos of BM, GS, and DBGP cryogel for hemolysis activity assay (Triton X-100 as positive control, and PBS as negative control).

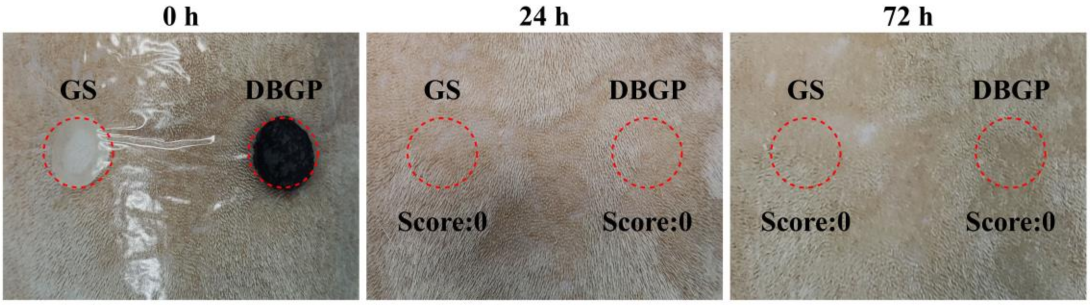

Figure S11. Digital photos of rat's skin irritated by GS and DBGP cryogel at 0, 24, and 72 h.

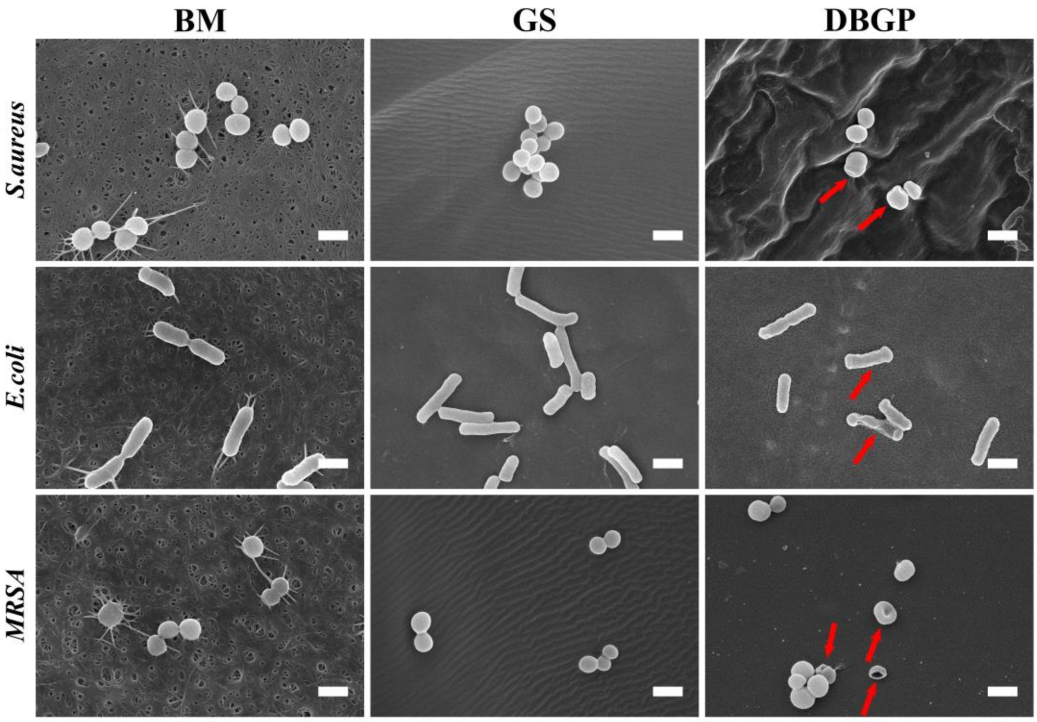

Figure S12. SEM images of *S. aureus*, *E. coli* and *MRSA* after co-culture with BM, GS, and DBGP cryogel (red arrows indicate bacterial deformation and death). Scale bar: 1 μm.

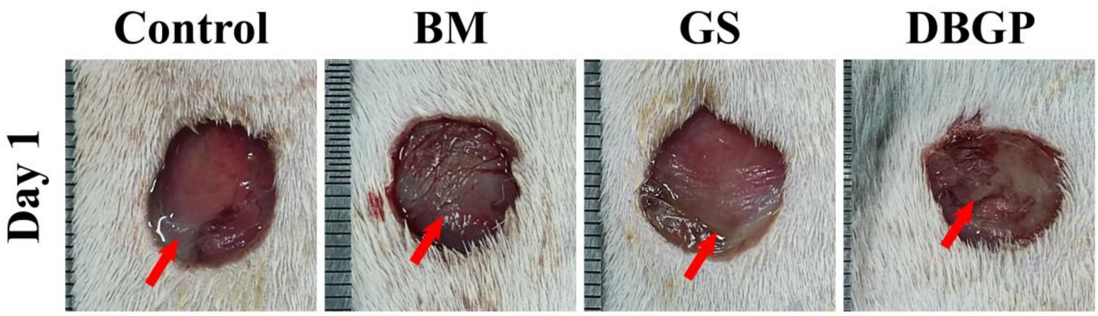

Figure S13. The digital photos of infected wounds with pus (indicated by red arrow) on day 1.

Table S1. Preparation parameters of DBGP cryogels.

**Cryogels**	**BC (wt.%)**	**Gel (wt.%)**	**18% DA (mL)**	**6% SP (mL)**
**DBGP**	0.325	4	0.6	1.2
**DBGP-1**	0.1625	4	0.6	1.2
**DBGP-2**	0.65	4	0.6	1.2
**DBGP-3**	0.325	4	0.4	0.8
**DBGP-4**	0.325	4	0.8	1.6
**DBGP-5**	0.325	2	0.6	1.2
**DBGP-6**	0.325	6	0.6	1.2

Table S2. Comparisons of swelling ratio (SR) and water vapor transmission rate (WVTR) between DBGP cryogel and reported wound dressings.

**Materials**	**SR (%)**	**WVTR ($g\ m^{-2}\ d^{-1}$)**	**References**
DBGP cryogel	3800	2368	This work
Kaltostat® alginate dressing	2472	870	[3]
Hydrosorb® hydrogel	1518	300	[3]
Mepilex® foam dressing	1124	620	[3]
C-OD_1 sponge	3200	3316.8	[4]
CM-CTS fabric	1250	2603 ± 34	[5]
SA-COS-ZnO hydrogel	150	682	[6]
aSS-PU_1 sponge	2453	2864	[7]
PVA/Starch/0.14GCN/0.7Ag@ TiO_2 NPs hydrogel	500	1008	[8]
PGC_4 film	800	2003 ± 26.4	[9]
PCA nanofiber membrane	62.5 ± 14.36	2500	[10]
50/50 PVA/SPI hydrogel	838.8 ± 12.3	2430.8 ± 77	[11]
70HPMC/30CMS/50ZnO-NPnanocomposite film	157.33 ± 2.08	178.05 ± 4.36	[12]

Table S3. Scoring criteria of the skin irritation test.

Skin response		Score
Erythema and escharosis		
	No erythema	0
	Mild erythema (barely visible)	1
	Obvious erythema (scattered or small erythema)	2
	Moderate to severe erythema	3
	Severe erythema (purplish red) to slight escharosis	4
Edematization		
	No edema	0
	Mild edema (barely visible)	1
	Mild edema (skin protuberance with clear boundary)	2
	Moderate edema (skin protuberance of about 1 mm)	3
	Severe edema (enlarged range of skin protuberance exceeding 1 mm)	4

Evaluation criteria of skin irritation intensity: weak irritation, 0-2 point(s); moderate irritation, 3-5 points; severe irritation, 6-8 points.

Table S4. Scores of skin irritation intensity in rats with different objects.

Test objects	24 h	72 h	Irritation intensity
GS	0	0	No irritation
DBGP cryogel	0	0	No irritation

Table S5. Primers used in the qRT-PCR analysis.

Genes	Forward primer	Reverse primer
iNOS	CCTGCTTTGTGCGAAGTGTC	CCCAAACACCAAGCTCATGC
ARG-1	ACATTGGCTTGCGAGACGTA	ATCACCTTGCCAATCCCCAG
IL-1β	TGCCACCTTTTGACAGTGATG	GTGCTGCTGCGAGATTTGAA
TNF-α	TAGCCCACGTCGTAGCAAAC	GCAGCCTTGTCCCTTGAAGA
CD86	CTTACGGAAGCACCCACGAT	CGGCAGATATGCAGTCCCAT
IL-10	CCAAGGTGTCTACAAGGCCA	GCTCTGTCTAGGTCCTGGAGT
CD206	GTGGGGACCTGGCAAGTATCCAC	CACTGGGGTTCCATCACTCC
CD163	AGACACACGGAGCCATCAAA	GGAGCGTTAGTGACAGCAGA
GAPDH	GGTTGTCTCCTGCGACTTCA	TGGTCCAGGGTTTCTTACTCC

References

[1] Y. Qian, Y. Zheng, J. Jin, X. Wu, K. Xu, M. Dai, Q. Niu, H. Zheng, X. He, J. Shen, *Adv. Mater.* **2022**, *34*, 2200521.

[2] X. Zhao, L. Wang, C. Tang, X. Zha, Y. Liu, B. Su, K. Ke, R. Bao, M. Yang, W. Yang, *ACS Nano* **2020**, *14*, 8793.

[3] M. Minsart, S. Van Vlierberghe, P. Dubruel, A. Mignon, *Burns Trauma* **2022**, *10*, tkac024.

[4] X. Wang, Q. Dang, C. Liu, G. Chang, H. Song, Q. Xu, Y. Ma, B. Li, B. Zhang, D. Cha, *Carbohydr. Polym.* **2022**, *277, 118782.*

[5] Z. Jiang, L. Li, H. Li, L. Xia, H. Hu, S. Wang, C. Liu, J. Chi, Y. Yang, F. Song, W. Liu, B. Han, *Carbohydr. Polym.* **2022**, *280, 119032.*

[6] M. Zhang, X. Qiao, W. Han, T. Jiang, F. Liu, X. Zhao, *Carbohydr. Polym.* **2021**, *266, 118100.*

[7] S. Chen, S. Li, Z. Ye, Y. Zhang, S. Gao, H. Rong, J. Zhang, L. Deng, A. Dong, *Chem. Eng. J.* **2022**, *446, 136985.*

[8] A. Ahmed, M. B. K. Niazi, Z. Jahan, T. Ahmad, A. Hussain, E. Pervaiz, H. A. Janjua, Z. Hussain, *Eur. Polym. J.* **2020**, *130,109650.*

[9] N. Rashid, S. H. Khalid, I. U. Khan, Z. Chauhdary, H. Mahmood, A. Saleem, M. Umair, S. Asghar, *Acs Omega* **2023**, *8*, 7575.

[10] J. Yin, L. Xu, A. Ahmed, *Adv. Fiber Mater.* **2022**, *4*, 832.

[11] N. Varshney, A. K. Sahi, S. Poddar, N. K. Vishwakarma, G. Kavimandan, A. Prakash, S. K. Mahto, *ACS Appl. Mater. Interfaces* **2022**, *14*, 14033.

[12] V. Pitpisutkul, J. Prachayawarakorn, *Carbohydr. Polym.* **2022**, *298, 120082.*
